# Characterization of More Than a Third of a Million Toy-Related Fractures

**DOI:** 10.5435/JAAOSGlobal-D-22-00013

**Published:** 2022-03-03

**Authors:** Scott J. Halperin, Sofia Prenner, Harold G. Moore, Jonathan N. Grauer

**Affiliations:** 1 From the Yale School of Medicine, New Haven, CT (Halperin); the Weill Cornell Medical College, New York, NY (Moore); and the Department of Orthopaedics and Rehabilitation, Yale School of Medicine, New Haven, CT (Grauer).

## Abstract

The toy industry has grown substantially over time, with billions of dollars of toys sold each year in the United States alone. Even after safety considerations, injuries can result. This study examined toy-related fractures in the US Emergency Departments (ED).

**Methods:**

The 1999 to 2018 National Electronic Injury Surveillance System from the US Consumer Product Safety Commission was examined data for fractures involving a toy-related injury. The incidence, trends, and anatomic locations for such fractures were assessed.

**Results:**

In total, 347,135 toy-related fractures were identified, of which 237,754 (68%) were in patients younger than 18 years, 182,516 (53%) were sustained by male subjects, and a 95% yearly incidence increase was observed over the years of the study. Anatomically, 37% were shoulder/arm/elbow, 24% wrist/hand/finger, 19% ankle/toe/foot, 10% leg/knee, 6% face/neck/head, and 4% trunk/pubic region.

**Discussion:**

Despite safety considerations with toy design, more than a third of toy-related fractures were seen in the ED, with a nearly doubling yearly incidence over the study period. This could be contributed to by increased production and prevalence of toys and/or rougher play and increased overall violence. These results are important not only for patient safety but also for orthopaedic surgeons, EDs, toy manufacturers, and policymakers.

The toy industry has grown substantially over the past decade, with revenue from toy sales rising significantly as the world economy develops. Between 2012 and 2014 alone, the US retail toy market revenue increased 4% (from $17.47 billion to $18.11 billion).^1^ In 2018, global toy retail sales surpassed $90.4 billion, while domestic US sales reached $21.6 billion.^2^ Online toy revenue has been rapidly increasing as well in the past 5 years, seeing an increase of 11.2% in revenue. In 2020, the online children's toy sales revenue was approximately $23.2 billion.^3^

With the increase in toy sales, toys are widely integrated into US homes and are a staple in family households. On average, an American child will receive more than $6500 worth of toys in their lifetime.^4^ From boats and blocks, to art and chemistry sets, to BB guns and boomerangs, and everything between, toys are ubiquitous in today's societies.

Injuries may occur secondary to a wide variety of interfaces with toys, including trauma secondary to a thrown toy, accidental injury from tripping over toys, and a host of other inadvertent interactions. Many toys have been studied because of the danger that they may pose to children. For example, modern toy guns available for purchase can paralyze, blind, and cause lasting injury to children.^5^ In fact, in 2011, a child was treated in a US emergency department (ED) for a toy-related injury every 3 minutes.^6^

Increasing toy sales poses a potential increased risk of injury to people of all ages. Of the many types of injuries that can occur, fractures are of great interest to the orthopaedic surgeon. This study aimed to examine toy-related fractures using the National Electronic Injury Surveillance System (NEISS) as a tool to analyze these injuries that result in ED visits.

## Methods

### Database/Study Population

NEISS is a database developed by the US Consumer Product Safety Commission. This tool, developed in 1972, publishes the most recent 20-year consumer product–related injuries resulting in a visit to the ED. The database uses information from more than 100 hospitals (including children's hospitals) of all sizes around the United States.

Each case has its unique validated weighing scale, with findings that can be extrapolated to national numbers. The results presented reflect these extrapolated findings. Data included were dates, narratives, locations of the injuries, the body parts injured, and the patient's sex, race, ethnicity, and age. To develop and record the necessary data, a coordinator is placed at each hospital.

Data of the NEISS database from 1999 to 2018 were used. Patients with injury related to a toy were identified based on the NEISS coding for toy products. The population was further narrowed to those who had sustained a fracture based on their diagnosis recorded in NEISS. Additional data elements abstracted included the year of injury, anatomic location of injury for all patients and subset of injures for patients younger than 18 years, and the type of toys.

### Statistical Analyses

Demographics, injury anatomic locations, hospital disposition, and trends of toy-related fractures leading to ED visits were assessed. For the larger pediatric set of injuries, toys were also grouped into six broad types (ball-related toys, riding toys, vehicle nonriding toys, weapon-related toys, arts-related toys, and other) for a subanalysis.

Information from the NEISS data set was extracted using code in STATA (Software for Statistics and Data Science). Data were then organized and used to create graphics using a combination of Excel and Prism. Studies based on the NEISS data set received exemption from review the author's institutional review board.

## Results

Based on the study inclusion criteria, 347,135 toy-related fractures were identified (Table 1). Of these, 237,754 (68%) were younger than 18 years (pediatric), and 182,516 (53%) were male subjects. Of note, in the adult patient population, 43,991 (60%) were women. In the pediatric patient population, 138,525 (58%) were boys.

**Table 1:** Demographic Information for Toy-Related Fractures in the ED Using NEISS From 1999 to 2018

	Adult	Pediatric	Total
Male	43,991	138,525	182,516 (53%)
Female	65,390	99,229	164,618 (47%)
Total	109,380 (32%)	237,754 (68%)	347,135

ED = Emergency Department, NEISS = National Electronic Injury Surveillance System

**Figure 1:**
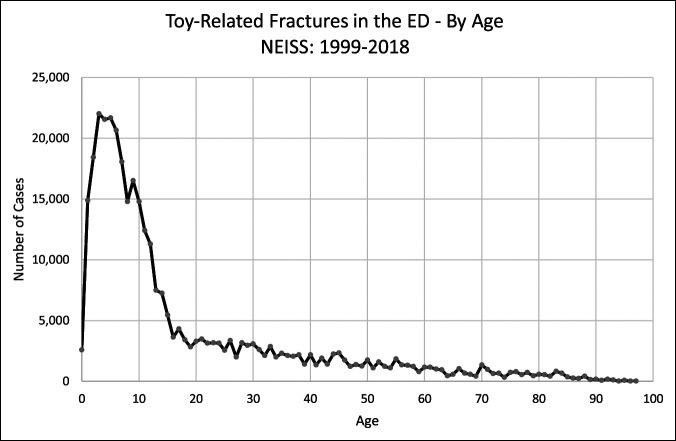
Graph showing toy-related fractures in the ED using data of NEISS form 1999 to 2018 by age. The results show that 68% of toy-related fractures occur in the pediatric population (younger than 18 years). ED = Emergency Department, NEISS = National Electronic Injury Surveillance System

Figure 1 depicts the age distribution of those sustaining toy-related fractures. Clearly demonstrated is the large pediatric peak, followed by a gradual decrease in incidence in the adult age range.

**Figure 2:**
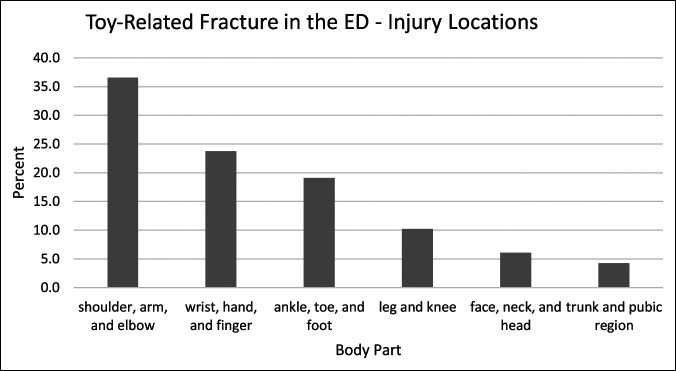
Graph showing toy-related fractures by locations using data of NEISS from 1999 to 2018. NEISS = National Electronic Injury Surveillance System

Anatomically, toy-related fracture injury locations in the total population were 37% shoulder/arm/elbow, 24% wrist/hand/finger, 19% ankle/toe/foot, 10% leg/knee, 6% face/neck/head, and 4% trunk/pubic region (Figure 2). In total, upper extremity injuries accounted for 61% of the injuries.

The disposition of these patients in the ED shows that approximately 90% were treated and/or discharged, 3% were transferred to another hospital, 7% were admitted within the same facility, and approximately 0.5% were not recorded or had another disposition.

**Figure 3:**
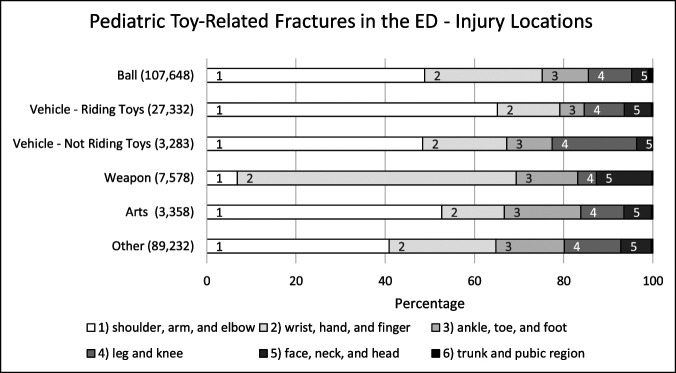
Bar graph showing pediatric toy-related fractures presenting to an ED and fracture locations based on their percentage of occurrence within a toy type group using data of NEISS from 1999 to 2018. This data set was separated into six broad groups including ball-related, riding-related, vehicle (not riding)-related, weapon-related, arts-related, and other causes. The number of fractures were quantified as seen in the parentheses in the x-axis. ED = Emergency Department, NEISS = National Electronic Injury Surveillance System

Because of the large peak of toy-related fractures in the pediatric population (representing 68% of the population), these were separately analyzed by toy type (Figure 3). Based on this analysis, the breakdown of injuries by toy type were ball-related (107,648 cases, 45%), riding-related (27,332 cases, 11%), vehicle not riding-related (3283 cases, 1%), weapon-related (7578 cases, 3%), arts-related (3358 cases, 1%), and other causes-related (89,232 cases, 38%) injuries. Shoulder/arm/elbow was the most common injury location in all groups, except in weapon-related toy fractures, where wrist/hand/finger was the most common injury location (63%).

**Figure 4:**
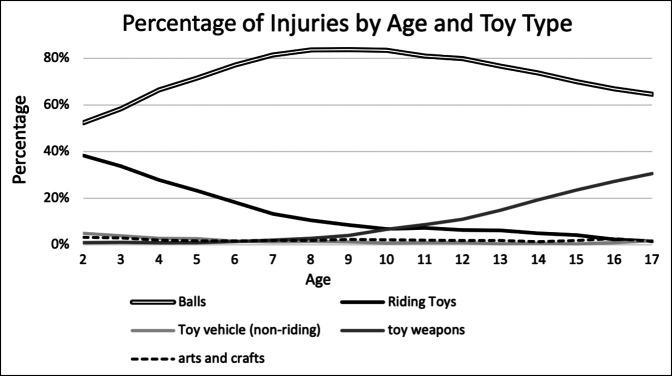
Graph showing pediatric trends in injuries due to toy types at different ages (excluding other toy type). Here, ball-related fractures were the most common throughout the pediatric years. Next, as pediatric patients get older, the prevalence for toy weapons increased prevalence, whereas that for riding toys decreased. Prevalence for arts-related and nonriding toy vehicles remained constant at a low percentage.

After analyzing the pediatric trends of toy types at different ages (excluding other toy type), ball related fractures were the most common throughout the pediatric years (Figure 4). As pediatrics got older, toy weapons increased in prevalence, while riding toys decreased in prevalence. Arts-related and non-riding toy vehicles remained fairly constant at a low percentage.

**Figure 5:**
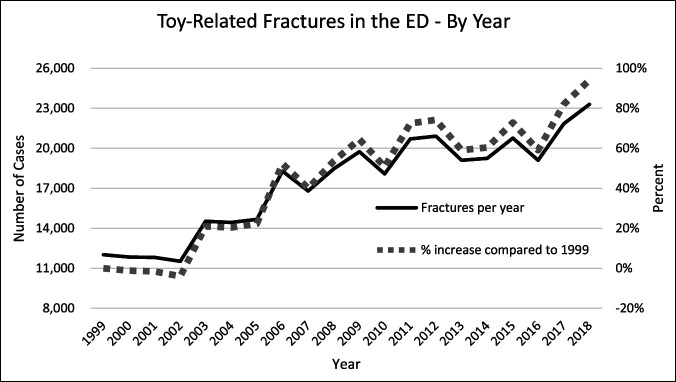
Graph showing toy-related fractures in the ED by year using NEISS. The primary x-axis shows the number of cases and the secondary x-axis the percentage increase compared with 1999. A 94% increase is seen in toy-related injuries from 1999 to 2018. ED = Emergency Department, NEISS = National Electronic Injury Surveillance System

In examining the number of toy-related fractures in EDs each year (Figure 5), a 94% increase (12,002-23,296 cases) was observed from 1999 to 2018. For context, during this period, the US population increased by just 17%, meaning the population-adjusted increase was 65%.

## Discussion

This study assessed the incidence of toy-related fractures presenting to the ED over 20 years by examining the NEISS database. This is a Consumer Product Safety Commission-established database of consumer product–related injuries resulting in a visit to the ED in the United States. Given the ongoing growth of the toy industry over the study period and the associated increased prevalence of toys that the population is exposed to, the authors examined toy-related fractures captured by this database over 20 years.

During the study period, 347,135 toy-related fractures were identified. With this, a 94% incidence in fractures increase was seen by year over the study period. Of note, even when correcting for the 17% increase in the US population over this period, a 65% increase was noted in population-adjusted incidence. This may be due to increased toys per capita and increased violence with play.

Most injuries occurred in children (68%) and male subjects (53%). The location of the fractures was predominantly in the upper extremities (37% shoulder/arm/elbow and 24% wrist/hand/finger), and 90% of subjects were discharged from their initial ED visit with or without the need for treatment. Interestingly, while 47% of the pediatric population sustained fractures were girls, this increased to 60% in the adult population. This increase in the adult female population is likely due to women being more likely to be the caregiver (75% of caregivers were women), making them more likely to interact with their children's toys.^7^

The increasing incidence of toy-related fractures is important to recognize from multiple perspectives. In light of these data, physicians in EDs may concentrate additional time in their history and physical examinations to toy-related injuries because these histories may be difficult to elicit, particularly with young children. Furthermore, physicians can look to advice their patients on safety when using various household toys. Adults constituted 32% of the study population, and ED physicians need to recognize this phenomenon in an older cohort whose age might make toy-related injuries lower on a differential diagnosis.

The increased incidence of toy-related fractures should also be recognized by the toy industry. Although the industry places significant time and attention on the safety of toy products, the increasing number of toy-related fractures is a public health concern, and the need for persistent diligence concerning toy safety should be recognized by manufacturers.

This study is limited by several factors. Importantly, this data set may underestimate the incidence of toy-related injury because many patients with fractures may not seek care in the ED. In addition, this study focused on fractures and did not assess other types of potential toy-related injuries.

Despite these limitations, this study clearly identifies an increasing incidence and prevalence of toy-related fractures in the general population over the 20 years studied. Recognition of this phenomenon is important for physicians in clinical settings, policymakers, and the toy industry itself because the exposure of the general population to toys seems increasing.

Future studies should investigate whether specific toys need to be considered from a safety perspective. Researchers should also look into toy injury rates beyond of EDs, including clinics and primary care visits. Overall, this study found a 94% increase in toy-related fractures that led to ED admission from 1999 to 2018, showing the need for increased safety precautions while using and playing with toys.
